# High Salt Intake and Atherosclerosis Progression—Not Only via Blood Pressure: A Narrative Review

**DOI:** 10.3390/nu17213464

**Published:** 2025-11-03

**Authors:** Stanisław Surma, Bogusław Okopień, Andrew J. Murphy, Maciej Banach

**Affiliations:** 1 Department of Internal Medicine and Clinical Pharmacology, Medical University of Silesia, 40-752 Katowice, Poland; bokopien@sum.edu.pl; 2 Department of Preventive Cardiology and Lipidology, Medical University of Lodz, 93-338 Lodz, Poland; maciej.banach@icloud.com; 3 Division of Immunometabolism, Baker Heart and Diabetes Institute, Melbourne, VIC 3004, Australia; andrew.murphy@baker.edu.au; 4 Faculty of Medicine, John Paul II Catholic University of Lublin (KUL), 20-950 Lublin, Poland; 5 Ciccarone Center for the Prevention of Cardiovascular Disease, Johns Hopkins University School of Medicine, Baltimore, MD 21287, USA; 6 Liverpool Centre for Cardiovascular Science (LCCS), Liverpool L14 3PE, UK

**Keywords:** salt, sodium, atherosclerotic cardiovascular disease, cardiovascular prevention, dietary risk

## Abstract

Excessive dietary salt intake remains a critical and underestimated global health concern, strongly associated with increased cardiovascular disease risk. While the relationship between salt and arterial hypertension is well established, accumulating evidence highlights additional, blood pressure-independent mechanisms linking high salt intake with the progression of atherosclerosis. Beyond its hypertensive effects, high dietary salt directly damages the vascular endothelium by disrupting the glycocalyx, reducing nitric oxide synthesis, and increasing endothelial stiffness and inflammation. Excess sodium also impairs glycosaminoglycan buffering capacity and promotes immune cell adhesion, even in normotensive individuals. Furthermore, salt-induced dysbiosis of the gut microbiota alters the metabolic and inflammatory environment, lowering beneficial short-chain fatty acids and increasing pro-atherogenic metabolites such as trimethylamine *N*-oxide. Recent findings also implicate salt-driven modulation of hematopoiesis via Th17 cytokines, which enhances the production of pro-inflammatory monocytes that accelerate plaque development. These findings support the notion that high salt intake may be an independent and modifiable residual risk factor for atherosclerotic cardiovascular disease. Reducing dietary sodium—particularly from processed foods—should therefore remain a central component of both primary and secondary cardiovascular prevention. Although the optimal range of salt intake remains under discussion, a moderate reduction to below 5 g/day is considered safe and beneficial.

## 1. Atherosclerotic Cardiovascular Disease

Atherosclerotic cardiovascular disease (ASCVD) comprise a group of conditions including ischemic heart disease (IHD), ischemic stroke, and peripheral artery disease (PAD) [1]. These disorders collectively constitute the leading source of cardiovascular deaths, being responsible for nearly two-thirds (67.3%) of all cardiovascular-related mortality [2]. From 1990 to 2019, global deaths due to IHD rose markedly, from 5.7 million to 9.14 million, and projections suggest that by 2050 this figure may double to approximately 20 million [3,4]. Population-level data further demonstrate a progressive increase in overall cardiovascular mortality: 12.1 million deaths in 1990, 18.6 million in 2019, with estimates of 20.5 million in 2025 and 35.6 million by 2050 [2,4]. The expanding global burden of cardiovascular disease (CVD) is expected to be driven mainly by ASCVD [4,5]. This adverse epidemiological trend is largely attributable to the high prevalence of established cardiovascular risk factors [2]. Among them, diet-related factors have consistently ranked second only to hypertension over the past several decades [2], and forecasts indicate that this hierarchy is unlikely to shift before 2050 [4]. The coronavirus disease 2019 (COVID-19) pandemic has further contributed to the deterioration of dietary practices—manifested by increased consumption of processed food, emotional and irregular eating patterns, and greater intake of sugar-sweetened beverages and alcohol—which may amplify the long-term risk of ASCVD [6,7,8].

The present narrative review seeks to deliver a contemporary overview of both traditional and novel biological pathways linking excessive dietary salt consumption to the advancement of ASCVD, with a specific focus on mechanisms independent of hypertension.

## 2. Salt Intake and ASCVD: Global Perspective

Dietary risk, most commonly including excessive salt intake, insufficient consumption of whole grains, and low intake of legumes, accounts for approximately 40% of cardiovascular deaths worldwide [9]. Dietary risk factors rarely act in isolation; smoking, low physical activity, psychological stress, and income inequalities synergistically modulate ASCVD risk [9]. Excessive salt intake is one of the most important modifiable risk factors for CVD, especially ASCVD. According to a comprehensive analysis by Nie et al., examining the global burden of disease attributable to high-sodium diets from 1990 to 2021, excessive salt consumption was responsible for approximately 1.89 million deaths annually, representing approximately 7% of all cardiovascular deaths. The highest burden occurred in countries of East Asia and Central Europe, where the traditional diet is characterized by a high proportion of added salt and processed foods [10]. Another study by Liang et al. showed that a high-sodium diet accounted for approximately 10% of the global burden of IHD. In developed countries, a significant decline in mortality due to this condition has been observed—by over 20% over three decades—which the authors attribute to intensive educational campaigns and the reformulation of low-sodium food products. Model estimates indicate that a global 30% reduction in salt intake by 2030, in line with the WHO target, could prevent up to 700,000 deaths annually from IHD [11]. A study by Mao et al. points in a similar direction, analyzing demographic and geographic differences in the burden of IHD attributed to a high-sodium diet. Approximately 62% of the burden was attributed to men, and the highest mortality rates occurred in the 60–69 age group. Countries with a higher socioeconomic development index (SDI) showed a faster decline in the burden owing to effective policies to reduce salt content in processed foods, mandatory sodium labeling, and health campaigns targeted at the adult population [12]. Experts point out that reducing salt intake is one of the cheapest, most effective, and most widely available public health interventions for ASCVD prevention. Reducing salt intake by just 1 g per day can reduce the risk of cardiovascular death by approximately 5% on a population-wide basis, and the benefits of such a reduction also occur in people without diagnosed hypertension. Examples from countries such as the United Kingdom, Finland, and Japan demonstrate that consistent salt reduction programs lead to long-term reductions in population blood pressure and a significant decline in the incidence of ASCVD [13]. A significant factor influencing the relationship between dietary salt intake and ASCVD is socioeconomic status (including lower knowledge of factors affecting health, fewer financial resources, poorer access to healthcare and lifestyle disease prevention programs). It has been shown that populations with lower socioeconomic status are characterized by a more frequent consumption of highly processed foods (the main source of salt in the diet), which contain high amounts of salt [14]. This is related to the fact that people with lower socioeconomic status benefit less from salt reduction programs [15]. This is confirmed by the results of a study by Yoon et al., which found that among low-income Black and White US residents, nearly 80% consumed sodium exceeding the current recommended daily amount (in the US, less than 2300 mg sodium per day), which was associated with 10% to 30% of CVD mortality in this population [16].

## 3. Level of Salt Intake and ASCVD Risk

Sodium occurs naturally in both plant- and animal-derived foods. It also constitutes the principal element of table salt (2.5 g salt = ~1 g sodium), which is commonly added to meals for flavoring and preservation. In contemporary diets, the predominant share of sodium intake originates from industrially processed foods (approximately 75%), while smaller contributions come from foods of natural origin (15%) and from discretionary salt used in cooking or at the table (10%) [17,18,19].

Between 1990 and 2019, cardiovascular deaths attributable to excessive dietary salt rose by 41.08% [20]. In 2019 alone, CVD caused 18.6 million deaths worldwide, of which 6.9 million were linked to diet-related factors and 1.72 million specifically to high salt intake [2,4,20]. Beyond cardiovascular causes, excessive salt consumption contributes to an estimated 3 million deaths annually [21]. A meta-analysis by Wang et al., including 616,905 participants, demonstrated that individuals with higher salt consumption had a 19% greater risk of developing CVD (RR = 1.19; 95% CI: 1.08–1.30). Moreover, cardiovascular risk increased incrementally by approximately 6% for each additional gram of sodium consumed per day (approx. 2.5 g of salt) [22]. A study of 243 prehypertensive patients followed for 4.53 years assessed the impact of dietary salt on the risk of cardiovascular events. It was found that patients consuming ≥6 g of salt/day had a 97% increased risk of CVD (HR = 1.97; 95% CI: 1.08–2.27). After multivariate analysis, a significant increase in risk was particularly evident in smokers, those with a family history of hypertension, and those with other risk factors for cardiovascular disease, such as male gender and being overweight [23]. Zhao et al., in a meta-analysis of 645,006 participants, analyzed 24 h urinary sodium excretion (in a person with normal kidney function, the amount of sodium excreted in urine is equal to the amount consumed—the body strives for sodium homeostasis) and showed that sodium excretion and CVD risk were associated linearly (each 1000 mg increase in sodium intake increased the risk of CVD by 4%; RR = 1.04; 95% CI: 1.01–1.07) [24].

Low, moderate and high sodium intake is considered to be <2.3 g/day (<5.75 g/day of salt), 2.3–4.6 g/day (5.75–11.5 g/day of salt) and >4.6 g/day (>11.5 g/day of salt), respectively [25].

Global sodium consumption substantially surpasses levels considered safe for health. Both the World Health Organization (WHO) and the European Society of Cardiology (ESC) recommend a daily maximum of 5 g of salt (equivalent to 2.5 g sodium) [17,18,26]. Current estimates indicate that the worldwide mean intake is more than double this threshold, averaging 10.8 g of salt per day [27]. Considerable variation in salt intake levels exists among countries: United States 8.9 g; Canada 9.1 g; Poland 11.1 g; Brazil 9.0 g; France 7.6 g; Italy 9.7 g; Spain 9.7 g; China 17.7 g; and Sweden 8.2 g [28]. The majority of nations exhibit moderate-to-high salt consumption [25], typically two- to three-fold above recommended limits [29]. Epidemiological evidence suggests that the lowest cardiovascular risk corresponds to an intake of 3–5 g of sodium per day, with ~4 g being optimal [30]. It should be emphasized, however, that these are observational study results that are not supported by interventional studies or current recommendations regarding acceptable level of salt intake. Numerous studies have demonstrated benefits of reducing salt intake. For example, in a UK Biobank cohort of 176,570 adults followed for nearly 12 years, Ma et al. found that participants who “usually,” “sometimes,” or “rarely/never” added salt to meals (compared to “always”) had 19% (HR = 0.81; 95% CI: 0.73–0.90), 21% (HR = 0.79; 95% CI: 0.71–0.87), and 23% (HR = 0.77; 95% CI: 0.70–0.84) lower risk of CVD, respectively [31]. A daily reduction of 2.5 g in salt intake corresponded to a 20% decrease in CVD risk (RR = 0.80; 95% CI: 0.66–0.97) [21].

In a modeling study, Hendriksen et al. estimated that reducing salt intake by 30% could lower stroke incidence by 6.4–13.5%, IHD by 4.1–8.9%, and all-cause mortality by 0.6–1.3%. Achieving the WHO-recommended level of <5 g salt per day was associated with even greater reductions: stroke by 10.1–23.1%, IHD by 6.6–15.5%, and mortality by 0.9–2.3% [32]. These findings prompt the question of whether “less is always better.” Evidence suggests that both very low (<3 g/day) and very high (>5 g/day) sodium intakes are linked to adverse outcomes, resulting in a U-shaped risk curve [30]. The meta-analysis of observational studies by Gradual et al. (274,683 participants) demonstrated a J-shaped association between sodium intake and both CVD and all-cause mortality, with risks elevated above 5 g/day and below 2.7 g/day, relative to moderate intake levels (2.7–5 g/day) [33]. Similarly, the PURE study reported a U-shaped relationship between urinary sodium excretion and CVD/mortality, with the lowest risk observed between 3–5 g/day [34]. A parallel pattern has also been noted for serum sodium concentrations, reinforcing the concept that sodium, as an essential nutrient, is expected to have a physiological “safe range” akin to other electrolytes [30]. Excessive restriction may stimulate activation of the renin–angiotensin–aldosterone system (RAAS), which itself is a well-established risk factor for CVD [30].

Other research has yielded somewhat different results. For example, a meta-analysis by Gan et al. including over 2 million individuals from 29 cohorts (with >66,000 CVD events) suggested that the lowest risk was associated with intakes between 1.7–2.3 g/day of sodium [35]. Nonetheless, caution is warranted when interpreting these findings. Measuring the effect of a single nutrient on CVD outcomes carries inherent limitations. The reference method—24 h urinary sodium excretion—was not uniformly applied; many studies relied on self-reported dietary intake, which is substantially less accurate [30]. Furthermore, extremely low-salt populations, such as some hunter–gatherer communities in isolated regions (e.g., the Amazon), cannot be used as the basis for recommendations. In these groups, sodium intake is poorly quantified and average life expectancy rarely exceeds 40 years, confounding meaningful extrapolation [30].

Another important limitation is that many studies have used 1–2 measurements of dietary salt intake. This does not allow for a precise analysis of the association between daily dietary salt intake and CVD risk (such an analysis does not take into account variability in dietary salt intake). It is practically impossible to conduct a multi-year study in which participants would consume the same daily diet with lower versus higher salt content.

There are a number of methodological limitations that mean that the observed U- and J-shaped relationships between dietary salt intake and cardiovascular outcomes should be interpreted with caution. Observational studies, which constitute the majority in the case of salt and CVD, by their nature do not allow for the demonstration of a causal relationship; they can only help formulate hypotheses [36]. Moreover, observational studies may be affected by reverse causality—for example, individuals with existing health conditions often reduce salt intake following medical advice, which can bias associations and make low sodium intake appear harmful [30,32,33,34]. Additionally, differences in sodium assessment methods significantly affect findings: some studies rely on dietary recall or food frequency questionnaires, which are prone to underreporting, while others use the gold standard of 24 h urinary sodium excretion [30,32,33,34]. Methodological limitations also exist when relying on the analysis of 24 h urinary sodium excretion. Popular formulas for estimating 24 h sodium excretion from a single urine sample—Kawasaki, Tanaka, and INTERSALT—have been shown to significantly distort results compared to actual 24 h measurements. These formulas systematically overestimate sodium excretion at low intakes and underestimate it at high intakes, leading to erroneous conclusions about the relationship between salt intake and blood pressure. The mean estimation errors were −10 mmol (−30 mg) for the Kawasaki formula, −45 mmol (−1035 mg) for the Tanaka formula, and −52 mmol (−1196 mg) for INTERSALT. When the relationship between sodium and systolic/diastolic blood pressure was analyzed, only the actual 24 h measurement showed a linear and positive relationship, whereas the formulas generated an artificial J- or U-shape, which may erroneously suggest that very low sodium intake is harmful. Therefore, full 24 h sodium measurements should be used for epidemiological analyzes and formulating health recommendations, not simplified estimation methods [37]. Estimating 24 h urinary sodium excretion based on mathematical formulas is imprecise because in real life, it is practically impossible to consume a diet with the same salt content every day. Furthermore, urinary sodium excretion, like many physiological processes, is subject to a circadian rhythm, which also prevents estimating daily excretion based on the analysis of a single urine sample [38]. Such methodological heterogeneity introduces variability that limits cross-study comparability and weakens the strength of public health recommendations. Indeed, different studies report a wide range of ‘optimal’ sodium intake levels: some suggest the lowest risk occurs between 3–5 g/day [29,32], others report a nadir at 1.7–2.3 g/day [35], and still others find increased risk at both high and low extremes of intake [32,33]. Moreover, a significant limitation of the interpretation of studies on the effect of excess salt on ASCVD is the lack of information on dietary potassium intake and other dietary patterns. Dietary potassium plays a well-documented role in the regulation of blood pressure. Elevated potassium intake has been shown not only to reduce blood pressure levels but also to mitigate the adverse cardiovascular consequences associated with high sodium diet [25,39]. Therefore, the daily intake of potassium should be taken into account in studies assessing the effect of excessive salt intake on CVD. A study by Ma et al., including 10,709 participants followed for 8.8 years, found that each 1000 mg increase in 24 h urinary sodium excretion was associated with an 18% increase in CVD risk (HR = 1.18; 95% CI: 1.08–1.29). Importantly, each 1000 mg increase in 24 h urinary potassium excretion reduced the risk of CVD by exactly 18% (HR = 0.82; 95% CI: 0.72–0.94). The results of this study emphasize the important role not only of maintaining optimal dietary salt intake, but also of potassium [40]. A randomized clinical trial by Ding et al., involving 5746 stroke patients followed for 61.2 months, demonstrated that a diet with sodium chloride substitution for potassium chloride was associated with a 14% reduction in the risk of recurrent stroke (RR = 0.86; 95% CI: 0.77–0.95) and a 12% reduction in the risk of death (RR = 0.88; 95% CI: 0.82–0.96). Furthermore, no significant risk of hyperkalemia was found in those substituting potassium chloride (RR = 1.01; 95% CI: 0.74–1.38) [41]. From the perspective of ASCVD risk, the DASH-sodium study by Knauss et al., including 412 participants, provided very significant results. It showed that compared to a high-sodium diet, participants consuming lower amounts of sodium had a 9.4% lower 10-year risk of ASCVD. Furthermore, dietary salt reduction combined with the DASH diet enhanced this effect, with a 14.1% reduction in 10-year ASCVD risk [42]. The results of this study clearly indicate that reducing dietary salt intake, optimally combined with improving other aspects of nutrition, has anti-atherosclerotic effects. The limitations discussed are reflected in the research results. For example, the most recent meta-analysis by Surma et al. did not demonstrate the influence of excessive dietary salt intake, assessed as low versus high intake or 24 h urinary sodium excretion, on arterial function parameters such as carotid intima-media thickness (cIMT), carotid-femoral pulse wave velocity (cf-PWV) and augmentation index (AIx). Analysis of the relationship between dietary salt intake and arterial function requires further studies, preferably with a longer intervention period and a more accurate assessment of the actual amount of salt intake [43]. In the context of demonstrating the progression of atherosclerosis, perhaps the duration of exposure to excessive amounts of salt in the diet should be longer than in the follow-up in many studies [43,44]. These discrepancies underscore the need for more standardized, prospective, and mechanistically informed studies to guide global dietary sodium guidelines more precisely.

Thus, the current state of knowledge allows us to state that excessive salt consumption is still a serious global health threat. Regardless of the discrepancies in research, reducing dietary salt intake by 30% or to the recommended 5 g per day allows for a reduction in the burden of ASCVD. Too much reduction in dietary salt may have the opposite effect and increase the risk of ASCVD and mortality.

## 4. Pathophysiological Mechanisms

Considering the existence of a probably U-shaped association between the level of salt consumed and the risk of ASCVD, it should be emphasized that the pressing public health problem is too much, not too little, salt consumption. The deleterious effects of excess dietary sodium are commonly attributed to the effect of sodium on blood pressure and risk of hypertension [30]. Indeed, salt reduction is particularly recommended for patients with hypertension. It is worth mentioning that the use of the dietary approaches to stop hypertension (DASH) diet with low sodium content (1500–2300 mg of sodium per day) is associated with a greater antihypertensive effect than the use of RAAS inhibitors [45]. The pathophysiological relationship between excessive dietary salt and CVD is, however, more complex and includes arterial hypertension, vascular endothelium damage, oxidative stress, hormonal disorders, inflammation, intestinal dysbiosis, and stimulation of the sympathetic nervous system (SNS) [46,47,48]. In an international survey conducted by Surma et al. on the knowledge of physicians (n = 313) about the effects of salt on health, it was found that most of them explained the increased risk of CVD caused by excess dietary salt by its effect on blood pressure (93%). Direct vasotoxicity and induction of inflammation were less frequently indicated by respondents (only 59% of respondents indicated direct vasotoxicity and 52% indicated inflammation). Only 42.2% of respondents clearly stated that excessive salt in the diet accelerates atherosclerosis [49]. The results of this study indicate that the knowledge of this important CVD risk factor is insufficient among doctors. It is worth emphasizing that it has been known for years that even in the absence of elevated blood pressure, high dietary sodium is associated with negative impacts on cardiovascular health. Increased sodium intake is related to increased risk of acute coronary events and cardiovascular mortality independent of blood pressure [30]. The study by Wuopio et al., covering 9623 participants, showed that higher dietary salt intake was associated with both coronary and carotid atherosclerosis, even in participants with normal blood pressure and without known CVD [50]. Additional evidence comes from the work of Ma et al., who analyzed data from 501,379 individuals enrolled in the UK Biobank. Their findings indicated that the habitual addition of salt to meals was linked to a substantially greater risk of premature cardiovascular mortality, ranging from 11% to 36%. Specifically, the risk was higher by 11% for those who salted food “usually” and by 36% for those who “always” added salt, compared with individuals who reported never or rarely salting meals [51]. In the study by Wang et al., Mendelian randomization (which facilitates causal inference) was used to assess the association between a genetic predisposition to salting and the risk of ASCVD. The results showed a significant increase in the risk of PAD and stroke, particularly ischemic stroke, in individuals with a greater genetic predisposition to salting [52].

Crucially, the association persisted even after multivariable adjustment for numerous confounders, including hypertension, which is most commonly linked to excess dietary salt intake. These findings suggest that adding salt to meals elevates cardiovascular mortality risk independently of blood pressure status [51]. This underscores the significance of sodium’s direct toxic impact on the vascular system, separate from its pressor effects. Put differently, sodium exerts vasotoxic properties even in the absence of hypertension.

### 4.1. Classic Mechanism: Salt → Arterial Hypertension → ASCVD

As already mentioned, the most well-known mechanism linking excessive dietary sodium with CVD involves the effect on blood pressure. In young people with normal kidney function, the magnitude of change in systolic and diastolic blood pressure associated with changes in dietary sodium consumption is a rather modest 1.9 and 0.4 mmHg per 2300 mg/day change in sodium, respectively, after adjustment for body mass index (BMI) and alcohol consumption [30]. In the case of chronically excessive sodium consumption, higher kidney function (hyperfiltration) but longitudinally with greater kidney function decline is observed [53]. This is associated with hyperfiltration and subsequent damage to the glomeruli [53]. In addition to salt, factors that increase hyperfiltration include a diet rich in animal protein, obesity, obstructive sleep apnea, etc. [54]. Impaired kidney function increases the adverse effect of excess dietary salt on blood pressure. A diet rich in sodium leads to stimulation of the SNS, RAAS, reduced nitric oxide (NO) production, and excessive mineralocorticoid receptor (MR) stimulation [55]. As a consequence, increased sodium and water retention (and thus increased extracellular volume) and vascular damage are observed, which leads to increased blood pressure [55]. In patients with chronic kidney disease (CKD), the risk of ASCVD (IHD, myocardial infarction, stroke) was shown to increase from a sodium intake level of 2 g/day [56].

**Figure 1:**
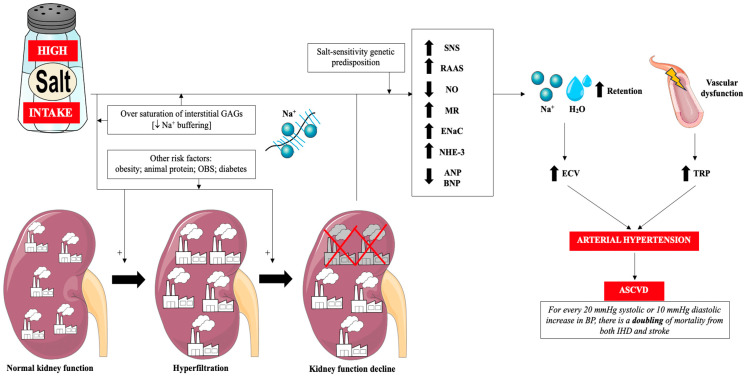
Classical mechanisms of the influence of excess dietary salt on the pathogenesis of hypertension and cardiovascular diseases. Prepared by the authors based on data discussed in the text, using elements from Servier Medical Art (https://smart.servier.com; accessed on 8 September 2025)—license: CC BY 4.0—no permission required. Abbreviations: Na^+^—sodium; GAGs—glycosaminoglycans; OBS—obstructive sleep apnea; RAAS—renin-angiotensin-aldosterone system; SNS—sympathetic nervous system; NO—nitric oxide; MR—mineralocorticoid receptor; ENaC—epithelial sodium channel; NHE-3—sodium-hydrogen antiporter 3; ANP—atrial natriuretic peptide; BNP—brain natriuretic peptide; H_2_O—water; ECV—extracellular volume; TPR—total peripheral resistance; ASCVD—atherosclerotic cardiovascular disease; IHD—ischemic heart disease; BP—blood pressure.

Glycosaminoglycans (GAGs) of the interstitial space play a very important role in the regulation of sodium management. Negatively charged GAGs constitute a buffer of positively charged sodium ions and thus protect the body against excessive increase in its concentration in serum. In other words, the degree of saturation of GAGs with sodium ions determines sodium sensitivity [57]. Sodium accumulating in GAGs in the interstitial space is gradually drained into the general circulation, when it does not cause an increase in blood pressure [57]. In subjects who chronically consume large amounts of sodium, the buffering capacity of GAGs is reduced and, as a result, there is a greater increase in blood pressure (sodium sensitivity) and greater exposure of endothelial cells (ECs) and kidneys to its increased concentration (and thus greater sodium toxicity to the vascular endothelium) [18,30,57,58]. It is worth mentioning that other mechanisms induced by excess salt in the diet also contribute to the development of arterial hypertension, including: reduced atrial natriuretic peptide/brain natriuretic peptide (ANP/BNP) production, overactive sodium-hydrogen antiporter 3 (NHE-3), overactive epithelial sodium channel (ENaC), disturbance to gut microbiota, enhanced systemic inflammation and alerted neurohormonal factors [59]. Figure 1 summarizes the classical mechanisms associated with excess salt in the diet and ASCVD via arterial hypertension.

### 4.2. Impact on Metabolic Disorders

Excessive salt intake is a significant factor in the development of metabolic disorders, including insulin resistance and glucose metabolism disorders, obesity, dyslipidemia, metabolic syndrome and metabolic dysfunction-associated steatotic liver disease (MASLD) [60,61,62,63,64]. All of these metabolic disorders accelerate the development of atherosclerosis [65]. A recently published study by Wuopio et al. demonstrated that salt consumption significantly regulate lipid and energy metabolism [66]. Higher salt intake is associated with adipose tissue hypertrophy, fat accumulation, stimulation of glucose conversion to fructose in the polyol pathway (resulting in hyperuricemia, increased insulin resistance, de novo lipogenesis, increased appetite, and induction of inflammation), increased ghrelin secretion (and stimulation of the appetite center), and a change in the nature of phosphatidylcholine and its derivatives to proatherosclerotic [66]. High sodium intake leads to excessive activation of the sympathetic nervous system and the RAAS, increased oxidative stress, and endothelial dysfunction, which impairs insulin signaling and glucose transport in skeletal muscle and adipose tissue [59]. Experimental studies have shown that high-sodium diets reduce insulin receptor substrate 1 (IRS-1) phosphorylation and glucotransporter 4 (GLUT4) translocation, and increase the secretion of proinflammatory cytokines such as tumor necrosis factor α (TNF-α) and interleukin 6 (IL-6), which exacerbates insulin resistance [59,67]. At the same time, intestinal dysbiosis induced by excess dietary salt (including a decrease in the level of *Lactobacillus* bacteria and an increase in the share of *Prevotella* and *Ruminococcaceae*) lead to increased intestinal permeability, chronic low-grade inflammation, adipose tissue dysfunction) which also contributes to further increases insulin resistance [59,67]. Importantly, studies indicate that both excessive and very low sodium intake can adversely affect insulin sensitivity—too little salt intake can compensatory activate the RAAS and sympathetic nervous systems, leading to increased insulin resistance [59]. This relationship is U-shaped, suggesting that moderate sodium intake is most beneficial in terms of the risk of metabolic disorders [59].

When considering the indirect pathways by which high dietary salt intake may accelerate atherosclerosis, the role of obesity deserves attention. Evidence indicates that individuals consuming excessive sodium face an elevated risk of becoming obese. For every additional gram of salt consumed daily, the likelihood of obesity increased by 28% in children (OR = 1.28; 95% CI: 1.12–1.45) and 26% in adults (OR = 1.26; 95% CI: 1.16–1.37) [68]. similarly, each 1-g/day rise in sodium intake was linked to a 24% higher risk of central obesity (OR = 1.24; 95% CI: 1.11–1.39) [69]. Therefore, limiting salt intake in the diet is an important element of obesity prevention [70]. This effect is related, among other things, to increased appetite and greater consumption of sweetened drinks [70]. This raises the question of whether high sodium intake reflects a broader pattern of unhealthy eating. Excessive salt consumption has been associated with multiple hormonal and metabolic disturbances that favor the development of obesity, including leptin resistance (leptin overproduction, fructose overproduction), ghrelin overproduction, dysregulation of adiponectin secretion, increased conversion of glucose to fructose in the polyol pathway, weakened lipolysis (decreased ANP and BNP levels), diminished neuropeptide Y, and increased cortisol secretion [61,62]. The aforementioned intestinal dysbiosis induced by excess dietary salt also promotes the development of obesity (e.g., by reducing the production of incretin hormones (glucagon-like peptide 1/2; GLP-1/GLP-2). Together these abnormalities favor positive energy balance and central adiposity, amplifying ASCVD risk [59,71].

Excess sodium also affects lipid metabolism. An increase in low-density lipoprotein (LDL) and triglyceride levels is observed, while a simultaneous decrease in high-density lipoprotein (HDL) is observed (e.g., by stimulating hepatic lipogenesis, activating RAAS, and disrupting bile acid circulation via the farnesoid X receptor- peroxisome proliferator-activated receptor α (FXR–PPAR-α) pathway, which leads to reduced cholesterol excretion) [59,66]. Furthermore, high salt intake may indirectly promote the development of a proatherogenic lipid profile through increased production of trimethylamine *N*-oxide (TMAO) and reduced short-chain fatty acids (SCFAs) concentrations [59,66].

A high-salt diet, by inducing the above-mentioned metabolic disturbances, also promotes the development of MASLD. Excessive salt intake has been shown to be causally associated with the occurrence of MASLD [64].

### 4.3. Impact on Endothelial Cells

A meal with a high salt content (6 g; e.g., pizza) leads to an increase serum sodium level by 3 mmol/L, which lasts for more than 4 h [72]. The observed increase in sodium was associated with an increase in systolic blood pressure by 6 mmHg [72]. A positive correlation was shown between the change in sodium and blood pressure (an increase in sodium by 1 mmol/L leads to an increase in systolic blood pressure by 1.9 mmHg) [72]. The question arises whether the increase in blood pressure after consuming a meal rich in sodium is associated with an increase in sodium or hypervolemia. In patients with CKD treated with renal replacement therapy, a hemodialysis procedure was performed with isovolaemia. The sodium concentration in the dialysis fluid was 145 versus 135 mmol/L. It was shown that the increase in blood pressure was caused by an increase in sodium [73]. A large amount of salt in the diet directly affects the vascular endothelium—sodium has a vasotoxic effect [17,18,46,74]. It has been shown that incubation of aortic endothelial cells (ECs) at sodium concentrations of 137 to 142 mmol/L (increase level by 5% in physiological concentration range) led to a 25% reduction in endothelial nitric oxide synthase (eNOS) activity. A 25% reduction in eNOS activity measurably reduces NO bioavailability, shifting the endothelial milieu toward vasoconstriction, leukocyte adhesion, and platelet activation—a canonical vasotoxic phenotype that accelerates atherogenesis [75]. It is worth mentioning here that there is an association between serum sodium level and CVD mortality. The lowest risk of death due to CVD is observed in people with sodium in the range of 135–141 mmol/L. Thus, serum sodium below 135 mmol/L associates with higher CVD mortality, likely reflecting combined effects of neurohormonal activation, comorbid burden, and endothelial stress in vulnerable patients [76].

It is appreciated that stiffening of ECs causes an increase in the resistance of blood vessels to blood pressure. Intertwined with vessel stiffness is the vascular endothelial glycocalyx, which is a gel layer composed of negatively charged GAGs with a thickness of 0.5 to 4.5 μm [18,46,77,78]. The vascular endothelial glycocalyx functions as an endothelial mechanoreceptor, ensuring proper reactions with morphological elements of blood and regulates its permeability [78]. Moreover, as a mechanotransducer, the glycocalyx mediates flow-dependent vasodilation by stretching the bilayer glycocalyx-lipid cytoskeleton and increases NO production by activating transient receptor potential (TRP) channels [79]. Proteoglycan glypican-1, not syndecan-1, plays a dominant role in propagating extracellular forces into the endothelial cells to activate signal transduction for the production of NO [79]. GAG chains (including heparin sulfate, chondroitin sulfate, and hyaluronan) are the main components of the glycocalyx and have a high negative charge, which makes them attractive to positively charged sodium ions flowing in the circulation [79,80]. The glycocalyx is a buffer that prevents excess sodium from passing from the blood into the tissues and enables efficient sodium excretion by the kidneys. The normal volume of the glycocalyx in the body, which is 1.7 L, allows for binding 7 g of sodium [81]. Syndecan-1 is an important element of the glycocalyx, and its increased concentration in the circulating system is a biomarker of glycocalyx damage [81]. It has been shown that increased serum sodium level within physiological limits is associated with increased syndecan-1 concentration in the circulation [82]. Increased sodium concentration leads to damage of the glycocalyx: reduced buffering properties, increased stiffness, and reduced height [79,80,83,84]. Damage to the glycocalyx increases sodium access to ENaC (ECs, as well as dendritic cells, macrophages), and intracellular sodium rapidly increases, which leads to increased endothelial stiffness and reduced eNOS activity [79,80,84]. Sodium overload transforms the ECs from a sodium release into a sodium-absorbing state [84]. Impairment of the buffering function of the glycocalyx and a reduction in its height leads to sodium accumulation in the body tissues (accumulation of excess sodium ions in the interstitial networks of GAGs, where sodium disrupts the function of GAGs and activates immune system cells) and the development of inflammation of the arterial wall, increased leukocyte adhesion, progression of atherosclerotic changes and a higher risk of thrombosis (increased adhesion of blood morphological elements to endothelial cells) [18,79,80,81,84,85]. Flow-mediated dilation (FMD) quantifies endothelium-dependent NO-mediated vasodilatation; lower FMD indicates impaired endothelial function and predicts adverse outcomes [86]. A clinical study has shown the occurrence of endothelial dysfunction measured by the brachial artery response to reactive hyperemia FMD 30 and 60 min after consuming a high-sodium meal [86]. This was also confirmed in other clinical studies [87,88].

Within the ECs, a submembrane zone with a thickness of several hundred nanometers (cell shell) is distinguished. In this cell shell, there is active monomeric (globular—G-actin) or fibrillar (F-actin) form [18,89,90]. G-actin is associated with lower stiffness, while F-actin with higher stiffness of the vascular endothelium [18,89,90]. It has been shown that sodium concentration >139 mmol/L leads to increased stiffness of the vascular endothelium [89]. It has been shown that a 5% increase in the plasma sodium level (sodium excess) stiffens ECs by about 25%, leading to cellular dysfunction [84]. This stiffness is associated with a decrease in eNOS activity, an increase in the number of ENaC, and an increase in the permeability of ECs [77]. A decrease in cortical stiffness due to F-actin depolymerization may increase the association of eNOS with G-actin and therefore directly stimulates NO release [91,92].

As previously mentioned, sodium saturation of GAGs reduces their buffering capacity and leads to increased exposure to the toxic effects of sodium on endothelial and kidney [18,57,63].

To translate experimental findings into clinical settings, future studies should integrate circulating syndecan-1 quantification, in vivo sublingual glycocalyx imaging, and sodium-sensitive MRI (^23^Na-MRI) to assess endothelial sodium accumulation and stiffness. Such multimodal approaches could validate salt-induced glycocalyx degradation and ENaC overactivation in humans and guide glycocalyx-protective therapeutic strategies [93,94,95].

Another important mechanism involved in direct vascular damage caused by excessive dietary salt intake are cardiotonic steroids, mainly marinobufagenin [96,97]. An increased level of marinobufagenin is observed in participants on a high-sodium diet. On the one hand, marinobufagenin increases natriuresis (via decrease NHE-3 activation), while, on the other hand, it causes vascular smooth muscle cell (VSMC) contraction and vascular stiffness and fibrosis. This is related to the effect of marinobufagenin on Na^+^/K^+^ ATP-ase and indirectly on Na^+^-Ca^2+^ exchanger (NCX), transforming growth factor -β1 (TGF-β1) and tyrosine kinase (Src) [96,97].

In the context of the direct vasotoxic mechanisms of excessive dietary salt, it is worth mentioning the results of the previously cited study by Wuopio et al. This study, which included participants from the Swedish SCAPIS cohort, demonstrated that even moderately elevated salt intake is associated with the development of metabolic signatures indicative of early, direct vascular damage. Individuals with a slightly excessive sodium intake had increased levels of phosphatidylcholine (PC) and lysophosphatidylcholine (LPC), membrane lipids with proinflammatory and prooxidant properties that promote endothelial dysfunction. The accompanying reduction in Krebs cycle metabolites suggests limited energy efficiency of vascular cells. The authors interpret these changes as a biochemical reflection of early vascular wall damage processes occurring with excessive salt intake, regardless of the effect on blood pressure [66].

**Figure 2:**
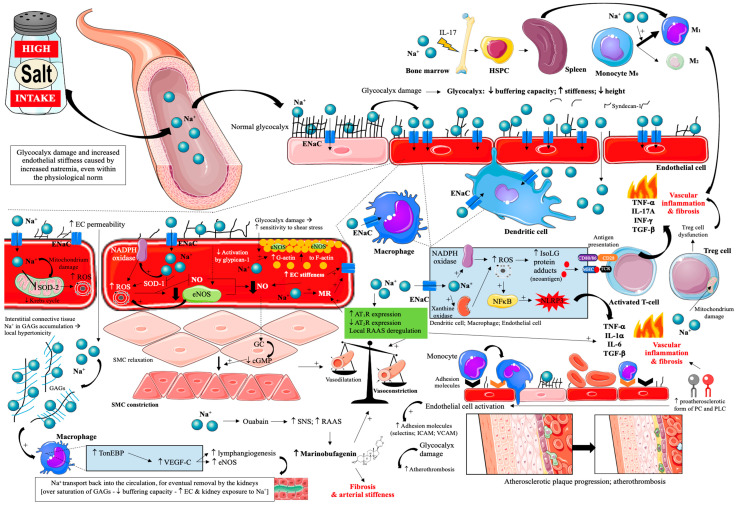
Effects of excessive dietary salt intake on glycocalyx and vascular endothelial cells. Prepared by the authors based on data discussed in the text, using elements from Servier Medical Art (https://smart.servier.com; accessed on 8 September 2025)—license: CC BY 4.0—no permission required. Abbreviations: Na^+^—sodium; ENaC—epithelial sodium channel; EC—endothelial cell; SOD 1/2—superoxide dismutase; ROS—reactive oxygen species; GAGs—glycosaminoglycans; NADPH oxidase—nicotinamide adenine dinucleotide phosphate oxidase; TonEBP—tonicity-responsive enhancer binding protein; SMC—smooth muscle cell; VEGF-C—vascular endothelial growth factor C; NO—nitric oxide; eNOS—endothelial nitric oxide synthase; MR—mineralocorticoid receptor; AT1R—angiotensin II receptor type 1; AT2R—angiotensin II receptor type 2; RAAS—renin-angiotensin-aldosterone system; ICAM—intercellular adhesion molecule; VCAM—vascular cell adhesion protein; NFκB—nuclear factor κB; IsoLG—isolevuglandins; NLRP3—inflammasome; TNF-α—tumor necrosis factor α; IL—interleukin; INF-γ—interferon γ; GC—guanylate cyclase; cGMP—cyclic guanosine monophosphate; CD—cluster of differentiation; MHC—major histocompatibility complex; TCR—T-cell receptor; PC—phosphatidylcholine; PLC—lysophosphatidylcholine.

Thus, the effect of a high-salt diet on vascular endothelial damage and atherosclerosis progression is very complex [17,18,72,73,74,75,76,77,78,79,80,81,82,83,84,85,86,87,88,89,90,91,92,93,94,95,96,97,98,99,100,101,102] and is summarized in Figure 2.

### 4.4. Impact on Gut Microbiota

Studies have clearly demonstrated gut dysbiosis in people with advanced atherosclerosis [103,104]. Moreover, a causal relationship between gut dysbiosis and lipid disorders has been demonstrated [105]. The important role of dysbiosis in the pathogenesis of metabolic disorders is confirmed by the results of interventional studies, which showed that the use of probiotics, prebiotics and synbiotics, by improving the composition of the gut microbiota, enhance lipid profile, reduced inflammation and oxidative stress in patients with IHD [106].

Based on the occurrence of key bacterial genera, three main enterotypes of the intestinal microbiota are distinguished: high in *Bacteroides* enterotype I, high in *Prevotella* enterotype II, and enterotype III characterized by a high level of *Ruminococcaceae* [107]. It has been shown that enterotype II was under-represented in IHD patients (*p* < 0.0001) compared to healthy individuals. In contrast, enterotype III was over-represented in IHD (*p* < 0.0001), confirming the appreciable differences in the gut microbiome between IHD patients and healthy subjects [107]. These findings should be interpreted as associations from cross-sectional data. Enterotype patterns are closely related to IHD status but do not establish causality.

Given the oral route of administration, it is not surprising that excessive dietary salt intake significantly affects the intestinal microbiota [108,109]. Increased salt content has been shown to cause intestinal permeability while altering the gut microbial composition, richness, and diversity (dysbiosis) [108,109]. One of the indicators of gut dysbiosis is the Firmicutes to *Bacteroidetes* (F/B) ratio. It has been shown that excessive dietary salt leads to an increased F/B ratio [109]. Moreover, exposure to high dietary salt results in a significant reduction in *Bacteroides* and an inverse increase in *Prevotella* and greater abundances of *Lachnospiraceae* and *Ruminococcaceae* [110]. Generally speaking, a high-salt diet leads to: a reduction in the number of lactate and butyrate-producing bacteria (including: *Lactobacillales*, *Leuconostocaceae*, *Basteroides fragilis*) and increase level of bacteria associated with inflammation and metabolic syndrome (including: *Lechnospiracaeae*, *Prevotellaceae*) [108]. All this leads to a disturbance of the physiological role of the gut microbiota and changes in the profile of the chemical substances produced by it: a decrease in the level of SCFA and GLP-1 and GLP-2, arachidonic acid, and an increase in the level of trimethylamine (TMA), glutamate and corticosterone. Moreover, lipopolysaccharide (LPS), indoxyl sulfate and pathogen-associated molecular patterns (PAMPs) as well as pathogenic bacteria themselves are introduced into the host organism [109,111,112]. Gut dysbiosis is closely related to the induction of inflammation, oxidative stress, proatherogenic effects and arterial hypertension [113,114]. In the previously cited study by Wuopio et al., it was shown that a high-salt diet led to increased levels of secondary bile acids, particularly deoxycholic acid (DCA) and lithocholic acid (LCA), known to exert cytotoxic effects on endothelial cells through activation of FXR and Takeda G protein-coupled receptor 5 (TGR5) receptors, leading to increased oxidative stress and mitochondrial dysfunction, were observed (most likely due to intestinal dysbiosis) [66].

In an experiment by Wilck et al., a high-sodium diet in mice led to the depletion of *Lactobacillus* bacteria (particularly *L. murinus*) and excessive activation of Th17 lymphocytes, accompanied by increased blood pressure and increased vascular inflammation. Returning to a diet with a normal sodium content resulted in the recovery of the *Lactobacillus* population, normalization of the immune response, and a reduction in the number of Th17 cells in the intestinal mucosa and blood. The authors also conducted a pilot study in humans (n = 12 healthy volunteers), in which a two-week increase in salt intake resulted in a decrease in the number of *Lactobacillus* bacteria in the feces, an increase in Th17 cell concentration in peripheral blood, and a moderate increase in blood pressure [115]. These results indicate that changes in the microbiota induced by excess salt are largely reversible upon returning to a lower-sodium diet, and the observed mechanism is also translational in humans. In a randomized, double-blind, crossover clinical trial by Chen et al. in 145 individuals, a moderate reduction in sodium intake (to approximately 2 g of sodium per day) resulted in increased concentrations of SCFAs—including butyrate and propionate, which are key metabolites of commensal bacteria that support intestinal barrier integrity, reduce inflammation, and improve metabolic sensitivity. Although this study did not directly analyze the composition of the microbiota, the results suggest improved metabolic and functional activity in response to sodium restriction [116]. In light of these data, it can be concluded that reducing salt intake favorably modulates the gut microbiota, restoring beneficial bacteria (*Lactobacillus*), increasing SCFA production, and suppressing pro-inflammatory immune system activity. This may be one of the links between a high-sodium diet and the development of atherosclerosis. The results of experimental studies indicate that the use of probiotics (especially those containing *Lactobacillus*) may alleviate gut dysbiosis induced by a high-sodium diet [117].

**Figure 3:**
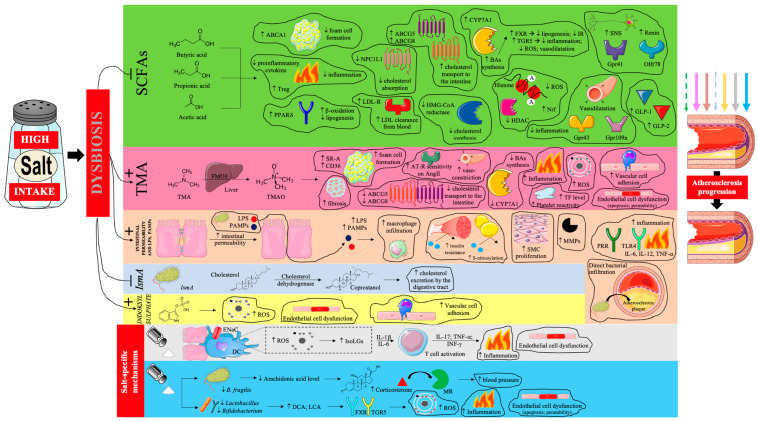
The influence of gut dysbiosis caused by excessive dietary salt intake on the progression of atherosclerosis. Prepared by the authors based on data discussed in the text, using elements from Servier Medical Art (https://smart.servier.com; accessed on 8 September 2025) —license: CC BY 4.0—no permission required. Abbreviations: SCFA—short-chain fatty acids; ABCA1—ATP-binding cassette transporter A1; PPARδ—peroxisome proliferator-activated receptor delta; NPC1L1—Niemann-Pick C1-Like 1; LDL-R—low-density lipoprotein receptor; LDL- low-density lipoprotein; ABCG5/8—ATP-binding cassette sub-family G member 5/8; CYP7A1—cholesterol 7 alpha-hydroxylase; BA—bile acid; FXR—farnesoid X receptor; IR—insulin resistance; TGR5—G-protein-coupled bile acid receptor; ROS—reactive oxygen species; HMG-CoA—3-hydroxy-3-methylglutaryl coenzyme A; HDAC—histone deacetylase; Nrf—nuclear factor erythroid–related factor; SNS—sympathetic nervous system; Gpr—G-protein-coupled receptor; Olfr—olfactory receptor; GLP—glucagon-like peptide; TMA—trimethylamine; FMO3—flavin-containing monooxygenase 3; TMAO—trimethylamine *N*-oxide; SR-A—scavenger receptors A; CD-36—cluster of differentiation 36; AT1R—angiotensin II receptor type 1; AngII—angiotensin II; TF—tissue factor; LPS—lipopolysaccharide; PAMPs—pathogen-associated molecular patterns; SMC—smooth muscle cell; MMPs—matrix metalloproteinases; PRR—pattern recognition receptor; IL—interleukin; TNF-α—tumor necrosis factor α; IsmA—intestinal steroid metabolism A; ENaC—epithelial sodium channel; DC—dendritic cell; IsoLGs—isolevuglandins; INF-γ—interferon γ; MR—mineralocorticoid receptor; DCA—deoxycholic acid; LCA—lithocholic acid; TGR5—Takeda G protein-coupled receptor 5. Dashed arrow—weakening of the antiatherosclerotic effect; solid arrow—intensification of the proatherosclerotic effect.

Thus, the proatherogenic effects of a high sodium diet, mediated by gut dysbiosis, are very complex [66,103,104,105,106,107,108,109,110,111,112,113,114,115,116,117,118,119,120,121,122,123,124,125] and are summarized in Figure 3.

### 4.5. Impact on Immune System

A recently described mechanism that may link excess dietary salt to ASCVD is dysregulation of the immune system. It has been shown that the amount of salt consumed, by inducing gut dysbiosis, significantly affects the activity of Th17 lymphocytes, which secrete cytokines IL-17 and IL-23, affecting bone marrow hematopoiesis [hematopoietic stem and progenitor cells (HSPC) migration to the spleen]. High-salt-induced haematopoietic activation promotes the expansion of inflammatory monocyte subsets, predominantly Ly6C^high^ in mice and their human counterparts CD14^++^CD16^+^ intermediate monocytes, characterized by enhanced NFκB signaling and increased secretion of IL-1β and TNF-α. These pro-inflammatory cells infiltrate atherosclerotic plaques and amplify atherosclerosis progression independently of blood pressure affects (Figure 2) [98]. Importantly, these hematological observations in mice were also seen in humans. Participants were given a diet containing 12 g of salt per day for ~50 days, then reduced to 9 g of salt per day for ~50 days, then to 6 g of salt per day for ~50 days, and finally returned to 12 g of salt per day for 30 days. Other macronutrients remained unchanged. With the reduction in salt intake, blood monocytes decreased, along with decreased levels of proinflammatory cytokines, particularly IL-23 and IL-17 in plasma. When higher levels of salt were reintroduced, blood monocytes increased again [98]. The results of this study are significant, causing inflammation and monocyte atherogenicity, which occur with the use of 12 g/day of salt, which is the intake of individuals in many countries around the world [18]. It is also possible to include the potential impact of excessive dietary salt intake on clonal hematopoiesis of indeterminate potential (CHIP), which is currently a novel risk factor for ASCVD. Although direct evidence is still limited, salt-induced inflammatory and haematopoietic activation might accelerate clonal expansion through chronic immune stimulation and oxidative stress [126]. An unhealthy diet that includes excessive amounts of salt has been shown to be associated with an increased incidence of CHIP [127]. However, we do note that mechanistic studies are required to understand if excessive salt does indeed impact clonal outgrowth.

Clinically, IL-17A, IL-23, soluble CD14 (sCD14), and circulating intermediate monocytes (CD14^++^CD16^+^) emerge as candidate biomarkers of salt-driven inflammatory haematopoiesis and vascular inflammation, enabling future risk stratification and therapeutic monitoring in high-salt exposure [128].

## 5. Conclusions and Clinical Implications

Given that the highest salt levels are found in processed foods and fast food, there is a need to further educate the public about healthy eating habits. Good examples include instant soups (one serving contains 1.9–2.4 g of salt), instant soups with large amounts of noodles (2.8–4.1 g of salt), pizza (7–12.8 g of salt), Chinese dishes (45.5–11 g of salt), kebabs (4–8.4 g of salt), and Indian dishes (3.6–6.1 g of salt; a typical fast food meal consisting of a sandwich, fries, and sauce contains about 4.5 g of salt, i.e., 90% of the recommended daily intake). Therefore, a number of food products can contain more than 5 g of salt per serving [90]. Current evidence demonstrates that in most parts of the world, daily salt intake exceeds recommended thresholds by two- to threefold. However, given the methodological limitations inherent in studies assessing sodium consumption and CVD risk, interpretations must be made with caution. The relationship between sodium intake and CVD appears to follow a U-shaped curve: while reduction in consumption is advisable, complete elimination is neither realistic nor desirable. Excess dietary salt accelerates atherosclerotic processes, not solely through blood pressure elevation (Table 1), but more importantly via direct endothelial toxicity. Even in individuals whose blood pressure does not rise with a higher sodium intake, excess salt damages the vascular lining through non-hypertensive mechanisms. Considering the pro-inflammatory properties of sodium, it may be appropriate to classify excessive intake as a residual risk factor for ASCVD. Preventive strategies should emphasize diets low in processed foods, which represent the major source of sodium in modern nutrition. Although excess salt is recognized as a conventional risk factor for CVD, ongoing efforts must prioritize educating healthcare professionals to improve awareness and attitudes regarding the vascular consequences of high dietary sodium (not only the risk of hypertension, but also CVD and death) [129]. Excessive salt intake should be considered a marker of an unhealthy diet, which contributes to the risk of many diseases. In the context of CVD, it is worth mentioning that excess dietary salt (and therefore an unhealthy diet) may contribute to the development of periodontitis, which is an increasingly well-documented risk factor for cardiovascular complications, including ASCVD [130,131,132].

However, it should be remembered that sodium reduction should be individualized, taking into account comorbidities. For example, the SODIUM-HF study did not demonstrate improved prognosis in heart failure patients following a diet with a sodium content of <1600 mg/day [133]. In patients with CKD, salt restriction is one of the pillars of nephroprotective management [134]. In healthy individuals, excessive sodium reduction can contribute to the stimulation of the sympathetic nervous system and the RAAS, consequently triggering various metabolic disorders. Importantly, cardiovascular protection results not only from sodium restriction but also from comprehensive dietary improvements, including increased potassium intake and improved overall dietary quality. Diets such as the DASH and Mediterranean diets—characterized by high amounts of fruits, vegetables, and minimally processed foods—have demonstrated synergistic effects on blood pressure, oxidative stress, and vascular function. Public health efforts should also focus on actively identifying individuals with very high sodium intake, particularly among those of lower socioeconomic status, who are more likely to consume inexpensive, highly processed foods high in sodium and low in potassium. Addressing these disparities through education, reformulation policies, and dietary counseling is essential for equitable prevention of salt-related atherosclerosis.

Finally, although ample mechanistic evidence supports the effects of salt on blood vessels, long-term, disease-specific intervention studies are still needed to precisely define sodium intake thresholds for different clinical and sociodemographic populations.

## Figures and Tables

**Table 1:** Summary of the proatherosclerotic properties of excessive dietary salt.

System/Compartment	Predominant Phenotype Under High Sodium	Dominant Biological Drivers	Salient Features/Readouts	Net Effect on Atherogenesis
**Hemodynamic regulation**	Sustained pressor load (arterial hypertension)	Renal sodium and water retention; SNS/RAAS activation; MR signalling; ↓ NO bioavailability; ENaC and NHE-3 overactivation; ↓ ANP and BNP levels	↑ TPR and vascular tone; endothelial vasoconstrictor bias	↑ Atherogenesis via pressure-mediated vascular injury
**Kidney**	Glomerular stress and injury	Chronic hyperfiltration; reduced GAG buffering; renal sodium overload	Glomerulosclerosis; declining filtration; CKD progression	Renal–vascular amplification of atherogenic risk
**Vascular endothelium—glycocalyx**	Glycocalyx thinning/fragmentation	Oversaturation of interstitial proteoglycans; sodium accumulation at the surface	↓ Buffering; ↑ exposure of EC to Na^+^; increased cell–blood element interactions	Loss of endothelial protection; thrombosis-prone interface
**Vascular endothelium—mechanics/ion channels**	Endothelial stiffening with ↑ ENaC expression	Mechanical shear sensitivity; altered sodium handling	Reduced NO generation; VSMC contraction bias; vasoconstriction	Impaired vasodilation; plaque-promoting hemodynamics
**Vascular endothelium—oxidative and immune signalling**	Pro-oxidative, pro-inflammatory signalling	ROS; IsoLG neoantigen formation; NFκB and NLRP3 activation	Leukocyte activation; cytokine release; fibrotic remodelling	Acceleration of vascular inflammation and lesion growth
**Vascular endothelium—adhesion and permeability**	Upregulated adhesion and barrier leak	↑ ICAM/VCAM/selectins; junctional disruption; ↑ proatherosclerotic PC and LPC form	Enhanced monocyte recruitment and transmigration	Foam cell formation and plaque expansion
**Vascular endothelium—mitochondria and survival**	Mitochondrial injury and apoptosis	Oxidative damage; energy stress	↑ Endothelial permeability; cell loss	Unstable endothelium; pro-atherogenic milieu
**Vascular endothelium—vasoactive systems**	Local neurohormonal dysregulation	RAAS imbalance; cardiotonic steroids (e.g., marinobufagenin)	Vasoconstriction, fibrosis, arterial stiffening	Adverse remodelling that favours atherosclerosis
**Interstitial matrix/proteoglycans**	Buffering capacity exhaustion	GAG oversaturation with sodium	Greater sodium delivery to EC, kidney, and other organs	↑ Sodium toxicity; amplification of vascular injury
**Gut microbiota—SCFA axis**	SCFA depletion and signalling loss	Diet–microbiome disruption under high salt	Reduced anti-inflammatory/antioxidant tone; BP dysregulation; altered lipid handling	Withdrawal of protective effects against atherogenesis
**Gut microbiota—TMAO pathway**	↑ TMAO generation	Microbial conversion of dietary precursors	Foam cell promotion; dyslipoproteinaemia; endothelial stress; prothrombotic tendency	↑ Plaque formation and event risk
**Gut barrier and translocation**	Increased intestinal permeability	LPS/PAMPs translocation; bacterial products	Systemic inflammation; insulin resistance; macrophage vascular infiltration	Inflammation-driven atherosclerosis
**Gut-derived/host metabolites**	Adverse metabolite signalling	IsmA (↓ coprostanol → ↓ cholesterol excretion); indoxyl sulfate; arachidonic acid/corticosterone → MR overactivation; ↑ DCA and LCA levels	Oxidative stress; endothelial activation/adhesion	Pro-atherogenic metabolic programming
**Innate/adaptive immunity—T cell axis**	Th17 skewing and Treg impairment	↑ IL-17/IL-23 signalling; mitochondrial stress in immune cells	Pro-inflammatory vascular milieu	Heightened vascular inflammation
**Hematopoiesis and clonal dynamics**	Myeloid-biased inflammation	CHIP-related mutations foster proinflammatory clones	Exaggerated monocyte output and activation	Chronic inflammatory drive to plaque growth
**Monocyte/macrophage polarization**	Shift toward M1-like phenotype	Sodium-driven innate immune reprogramming	Amplified vascular inflammation and foam cell formation	Lesion progression and destabilisation

Abbreviations: ASCVD—atherosclerotic cardiovascular disease; BP—blood pressure; CHIP—clonal hematopoiesis of indeterminate potential; CKD—chronic kidney disease; EC—endothelial cell; ENaC—epithelial sodium channel; GAGs—glycosaminoglycans; HR—hazard ratio; ICAM—intercellular adhesion molecule; IL—interleukin; IsoLG—isolevuglandins; LPS—lipopolysaccharide; MR—mineralocorticoid receptor; NFκB—nuclear factor kappa B; NLRP3—NOD-like receptor family pyrin domain-containing 3 (inflammasome); NO—nitric oxide; OR—odds ratio; PAMPs—pathogen-associated molecular patterns; RAAS—renin–angiotensin–aldosterone system; ROS—reactive oxygen species; SCFA—short-chain fatty acids; SNS—sympathetic nervous system; TMAO—trimethylamine *N*-oxide; TPR—total peripheral resistance; Th17—T helper 17 cell; Treg—regulatory T cell; VCAM—vascular cell adhesion molecule; VSMC—vascular smooth muscle cell; ENaC—epithelial sodium channel; NHE-3—sodium-hydrogen antiporter 3; ANP—atrial natriuretic peptide; BNP—brain natriuretic peptide; PC—phosphatidylcholine; PLC—lysophosphatidylcholine; DCA—deoxycholic acid; LCA—lithocholic acid

## Data Availability

Not applicable.

## Abbreviations

The following abbreviations are used in this manuscript:

ASCVD	Atherosclerotic cardiovascular disease
IHD	Ischemic heart disease
PAD	Peripheral artery disease
CVD	Cardiovascular disease
COVID-19	Coronavirus disease 2019
RR	Relative risk
95% CI	95% confidence interval
WHO	World Health Organization
ESC	European Society of Cardiology
HR	Hazard ratio
RAAS	Renin-angiotensin-aldosterone system
DASH	Dietary approach to stop hypertension
cIMT	Carotid intima-media thickness
cf-PWV	Pulse wave velocity
AIx	Augmentation index
SNS	Sympathetic nervous system
MR	Mineralocorticoid receptor
CKD	Chronic kidney disease
GAGs	Glycosaminoglycans
ECs	Endothelial cells
OR	Odds ratio
eNOS	Endothelial nitric oxide synthase
NO	Nitric oxide
TRP	Transient receptor potential
ENaC	Epithelial sodium channel
SCFA	Short-chain fatty acid
TMA	Trimethylamine
PAMPs	Pathogen-associated molecular patterns
BMI	Body mass index
NHE-3	Sodium-hydrogen antiporter 3
MASLD	Metabolic dysfunction-associated steatotic liver disease
IRS-1	Insulin receptor substrate 1
GLUT-4	Glucotransporter type 4
TNF-α	Tumor necrosis factor α
IL-6	Interleukin 6
GLP-1/2	Glucagon-like peptide ½
LDL	Low-density lipoprotein
HDL	High-density lipoprotein
FXR	Farnesoid X receptor
PPARα	Peroxisome proliferator-activated receptor α
SDI	Socioeconomic development index
TRP	Transient receptor potential channel
TMAO	Trimethylamine *N*-oxide
FMD	Flow-mediated dilation
VSMC	Vascular smooth muscle cell
NCX	Na^+^-Ca^2+^ exchanger
TGF-β1	Transforming growth factor β1
Src	Tyrosine kinase
PC	Phosphatidylcholine
LPC	Lysophosphatidylcholine
DCA	Deoxycholic acid
LCA	Lithocholic acid
TGR5	Takeda G protein-coupled receptor 5
LPS	Lipopolysaccharide
IL-17	Interleukin 17
IL-23	Interleukin 23
HSPC	Hematopoietic stem and progenitor cells
NFκB	Nuclear factor κ B
IL-1β	Interleukin 1β
CHIP	Clonal hematopoiesis of indeterminate potential
